# Performance of variable selection methods using stability-based selection

**DOI:** 10.1186/s13104-017-2461-8

**Published:** 2017-04-04

**Authors:** Danny Lu, Aalim Weljie, Alexander R. de Leon, Yarrow McConnell, Oliver F. Bathe, Karen Kopciuk

**Affiliations:** 1 Sick Kids Research Institute, 555 University Avenue, Toronto, ON M5G 1X8 Canada; 2 Department of Systems Pharmacology and Translational Therapeutics, Perelman School of Medicine, University of Pennsylvania, 10-113 Translational Research Center, 3400 Civic Center Blvd, Bldg 421, Philadelphia, PA 19104 USA; 3 Department of Mathematics and Statistics, University of Calgary, 2500 University Drive NW, Calgary, AB T2N 1N4 Canada; 4 Department of Surgery, University of British Columbia, 950 West 10th Avenue, Vancouver, BC V5Z 1M9 Canada; 5 Department of Oncology, Tom Baker Cancer Centre, University of Calgary, 1331-29th St NW, Calgary, AB T2N 4N2 Canada; 6 Department of Surgery, University of Calgary, 3330 Hospital Drive NW, Calgary, AB T2N 4N1 Canada; 7 Cancer Epidemiology and Prevention Research, Alberta Health Services, 2210 – 2 Street SW, Calgary, AB T2S 3C3 Canada

**Keywords:** Stability-based variable selection, False discovery rate (FDR), High-dimensional biological data, Partial area under the receiver-operating characteristic curve (pAUC), Variable importance in projection (VIP)

## Abstract

**Background:**

Variable selection is frequently carried out during the analysis of many types of high-dimensional data, including those in metabolomics. This study compared the predictive performance of four variable selection methods using stability-based selection, a new secondary selection method that is implemented in the R package *BioMark*. Two of these methods were evaluated using the more well-known false discovery rate (FDR) as well.

**Results:**

Simulation studies varied factors relevant to biological data studies, with results based on the median values of 200 partial area under the receiver operating characteristic curve. There was no single top performing method across all factor settings, but the student *t* test based on stability selection or with FDR adjustment and the variable importance in projection (VIP) scores from partial least squares regression models obtained using a stability-based approach tended to perform well in most settings. Similar results were found with a real spiked-in metabolomics dataset. Group sample size, group effect size, number of significant variables and correlation structure were the most important factors whereas the percentage of significant variables was the least important.

**Conclusions:**

Researchers can improve prediction scores for their study data by choosing VIP scores based on stability variable selection over the other approaches when the number of variables is small to modest and by increasing the number of samples even moderately. When the number of variables is high and there is block correlation amongst the significant variables (i.e., true biomarkers), the FDR-adjusted student *t* test performed best. The R package *BioMark* is an easy-to-use open-source program for variable selection that had excellent performance characteristics for the purposes of this study.

## Background

Variable selection is an important first step in the analysis of diverse chemical data, where often the goal is to identify a subset of measured variables that can distinguish between two or more different groups. Including all measured variables is impossible in practice and leads to reduced precision of model estimates and over-fitting in most analytical methods [1]. Many variable selection methods have been developed for high- and ultra high-dimensional data settings and for a variety of modelling approaches and data types [2–4].

The R package *Biomark* [5, 6] includes these popular variable selection methods: student *t* test, Variable Importance in Projection (VIP) scores [7, 8] from Partial Least Squares Regression (PLS-DA) models, Least Absolute Shrinkage and Selection Operator (LASSO) [9], and Elastic Net [10, 11]. Each method has different strengths and weaknesses for identifying significant variables often found in biological data like in metabolomics, and possibly, for modelling them. Models for such data should be able to handle multi-collinearity in the measured variables, small *n*-large *p* cases (i.e., more variables than samples), sparsity (i.e., few significant variables), and multiple variables in a regression context, and should have ease of interpretation.

Resampling approaches used for prediction with variable selection methods tend to perform poorly when the numbers of samples within groups are small. Stability-based selection is a new general approach that can be used with several different analytical methods, from student *t* tests to various regression techniques [12, 13]. It is similar to multiple-testing methods like the FDR [14] and *q*-values [15, 16], as these approaches all employ secondary selection based on an initial evaluation of the variables. Stability-based selection operates by repeatedly taking subsets of variables and samples from the full data set, and then estimates and ranks the coefficients, scores or *P* values as generated by the chosen analytical method in each perturbed dataset. If the fraction of the time a variable is included in a fixed number of top variables in the perturbed datasets is high, it is deemed to be stable and, therefore, a potential significant variable (i.e., a biomarker). Those variables appearing by chance in a few perturbations will not be a consistent indicator of class differences when the results are averaged, so are not selected. Thus, stability-based selection, like the jackknife [17] approach, perturbs the data to identify those variables that are consistently selected as group difference indicators [5, 12, 13] to improve prediction.

Performance of the variable selection methods using their selected significant variables can be evaluated using Receiver Operating Characteristic (ROC) curves when the real significant variables (i.e., true biomarkers) are known, such as with simulated and spiked-in data. ROC curves are generated by starting with the first selected biomarker then sequentially including the remaining ones and plotting the proportion of false positives (*x*-axis) against the proportion of true positives (*y*-axis) at each step. The Area Under the ROC curves (AUC) summarizes the performance of the selected set of significant variables by a variable selection method on a scale between zero and one; that is, the AUC measures how well a random pair of samples, one from each group, is correctly classified. The higher the AUC, the better the biomarker classification method performs. Instead of calculating the AUC for the whole curve, often the partial AUC (pAUC) is calculated [18–20] since most of the true significant variables are usually selected first without too many false ones. Restricting the calculation of the AUC to a smaller range of values of the false positive rate (i.e., higher specificity) is appropriate for diagnostic and other medical tests based on biomarkers for use in a clinical situation [21] and is the metric adopted here.

The objective of this study was to evaluate the performance of four popular variable selection methods using the robust stability-based selection criterion and two of these methods (VIP and student *t* test) with an FDR adjustment to identify significant variables. Our evaluation metric for each method was the pAUC, which assesses predictive performance using model-based simulated biological data for each of the variable selection methods.

## Methods

### Simulation study design

To evaluate the variable selection methods, we used model-based simulated data that mimicked biological data that have undergone pre-processing and pre-treatment steps in a metabolomics analysis pipeline [22]. By varying several biological and experimental factors likely to impact variable selection, we could systematically evaluate their effects across the methods we considered, since we knew the identity of the true significant variables.

Metabolomics data are typically right skewed and their range can vary substantially between individual metabolites. Logarithmic or other transformations are used along with centering and scaling the data to unit variance before analysis [22]. The simulated datasets were generated assuming these pre-analytical steps have been followed, resulting in standard multivariate normal distributions, which is the joint distribution of correlated univariate normal variables with zero means and unit variances (i.e., scaled data). The parameters that were varied included the combined study sample size N = 50, 100; the number P = 50, 200, 1000 of measured variables; the percentage Q = 10, 15, 20% of significant variables; the effect size (or mean abundance or signal) Δ = 0.2, 0.4, 0.8 in the treatment (or disease) group; and the correlation structure. Similar to Wehrens et al. [13], the following correlation structures were adopted: (1) independent (i.e., pair-wise and higher order correlations were zero), (2) block correlation (i.e., correlation between significant variables was 0.7, between non-significant variables was 0.1, and between blocks was zero), and (3) autoregressive of order 1 [AR(1), with correlation rho^abs(i−j)^ between variables i and j, for rho = 0.5].

Combinations of the various parameter values resulted in a total of 36 distinct parameter configurations for each correlation structure. For each configuration, 200 datasets were simulated and results are based on medians across these datasets. For LASSO and Elastic Net, the mixing parameter α was set at 1 and 0.5, respectively, and the value of the regularization parameter λ was chosen when the number P × Q of significant variables was first selected or the maximum number of variables when fewer were identified. Two components were adopted for PLS-DA models and the *BioMark* default values for the percentage of variables (variable.fraction) included in the subgroups as well as percentage of samples removed per group (oob.size) were used. A default top fraction ntop = 10 and a stringent consistency threshold level min. present = 0.5 (i.e., 50%) [13] were used so that a selected variable had to be in the top 10 variables in at least half of the 200 resampled datasets. In every setting, two groups of equal sample sizes were used to compare the ability of the four stability-based and two FDR-adjusted selection methods to correctly identify the significant variables associated with the treatment group. The pAUC was set at 0.2, which corresponds to a false positive fraction of 0.2 or equivalently, a specificity of 80%. The R code used to generate these data and the results can be found here: https://people.ucalgary.ca/~kakopciu/Simulated Metabolomics Biomarkers R Code.docx.

### Spiked-in metabolomics dataset

The spiked-in dataset was from a study that developed a standardized approach to untargeted metabolomics biomarker discovery [23]. Standardized human serum samples from the National Institute for Standards and Technology (NIST) were spiked at physiologic concentrations (50-300 um) with two sets of distinct metabolites. Maximum spiked concentration was calculated at double the target concentration, with dilution sets of samples of 0.33 to twofold concentration differences prepared. Sample extraction and derivatization followed our standard laboratory protocols and each sample underwent gas chromatography-mass spectrometry (GC–MS) on a Waters Technology machine. All samples were randomly ordered and run on the same day. A total of 18 replicates from each of the two solution sets were compared to each other. Spiked-in metabolites from Solution 1 included glycine (Gly), serine (Ser), threonine (Thr), and aspartic acid (Asp) while those from Solution 2 included alanine (Ala), valine (Val), lysine (Lys), and pyroglutamic acid (Pyr). Table 1 provides details on the concentration ranges. Fifty-two other metabolites were included as all samples could be measured, i.e., were above the detection limits. Additional details on our experimental protocol and the dataset used in this study are available here: https://people.ucalgary.ca/~kakopciu/Steps in designing and preparing the spiked-in Data set.pdf and https://people.ucalgary.ca/~kakopciu/Spiked-in Data Set BMC Notes paper.csv, respectively.

**Table 1:** Reference and measured identification parameters for spiked metabolites, with range of serum concentrations and injected amounts over the dilutional series

Metabolite species	Concentration range (μM)	Injection amount range (ng)	GOLM database		Measured parameters		
			RI^a^	Select *m*/*z* ions	Mean RI	*m*/*z* ions	RSD^a^ (%)
*Solution 1*							
Glycine (3TMS)^a^	200–300	0.6–3.7	1302.7	174|248|276|100|86	1305.7	174|248|147	101
Serine (3TMS)	100–150	0.4–2.6	1352.8	204|218|278|306|100	1357.9	204|218|147	106
Threonine (3TMS)	80–120	0.4–2.4	1377.2	219|291|218|117|320	1382.9	291|218|117	122
Aspartic acid (3TMS)	26.5–41	0.1–0.8	1511.2	232|218|306|202|334	1512.7	232|218|100	70
*Solution 2*							
Alanine (2TMS)	187	0.6–3.7	1108.6	116|190|218|100|233	1098.9	116|204|118	100
Valine (2TMS)	50–300	0.5–2.9	1207.1	144|218|156|246|100	1209.9	144|218|72	116
Lysine (4TMS)	33–200	0.4–2.4	1881.2	156|174|317|230|434	1915.1	156|174|317	94
Pyroglutamic acid (2TMS)	8–50	0.1–0.5	1650.4	156|258|230|140|273	1516.6	156|258|147	20

^a^ *RI* Retention index, *RSD* relative standard deviation, *TMS* trimethylsilyl groups

## Findings

**Fig. 1:**
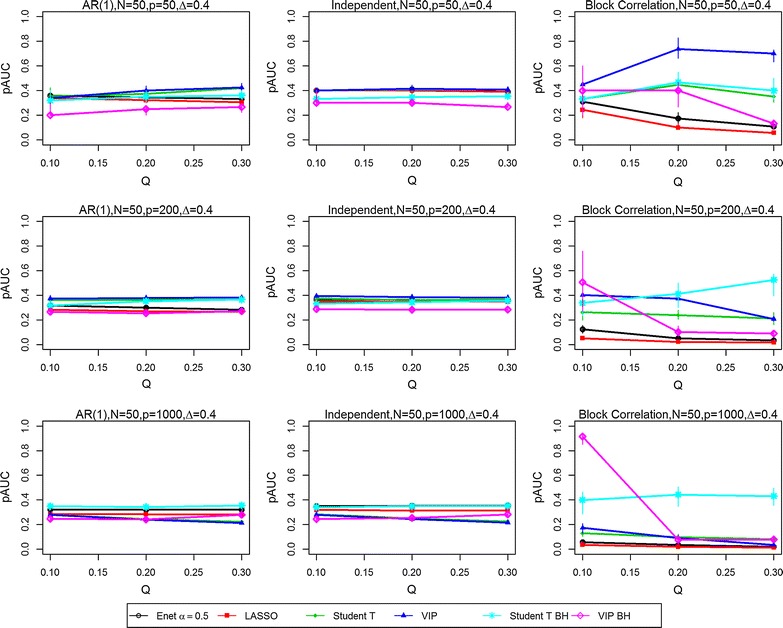
Partial Area Under the ROC curves (pAUCs) as the proportion of significant variables is increased from 0.1 to 0.3. Results are presented for all three correlation structures [independent, AR(1) and block correlation] for the six selection methods [four stability-based methods: Elastic Net (Enet), LASSO, student *t* test, VIP, and two FDR-adjusted methods: student *t* test BH and VIP BH]. The total number N of samples is 50 and the effect size Δ is 0.4. The total number P of variables is 50 (*Top row*), 200 (*Middle row*) and 1000 (*Bottom row*)

**Fig. 2:**
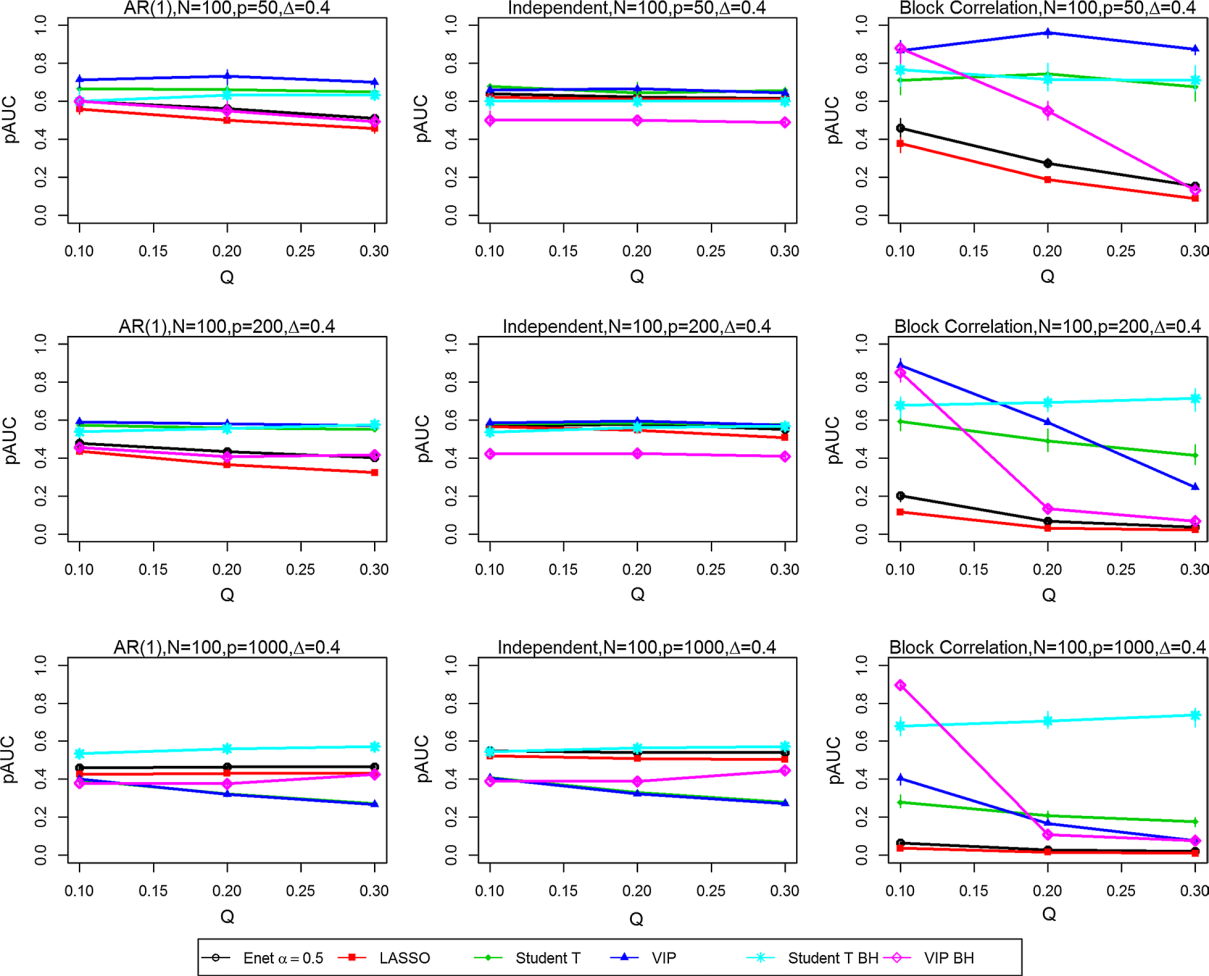
Partial Area Under the ROC curves (pAUCs) as the proportion of significant variables is increased from 0.1 to 0.3. Results are presented for all three correlation structures [independent, AR(1) and block correlation] for the six selection methods [four stability-based methods: Elastic Net (Enet), LASSO, student *t* test, VIP, and two FDR-adjusted methods: student *t* test BH and VIP BH]. The total number N of samples is 100 and the effect size Δ is 0.4. The total number P of variables is 50 (*Top row*), 200 (*Middle row*) and 1000 (*Bottom row*)

**Fig. 3:**
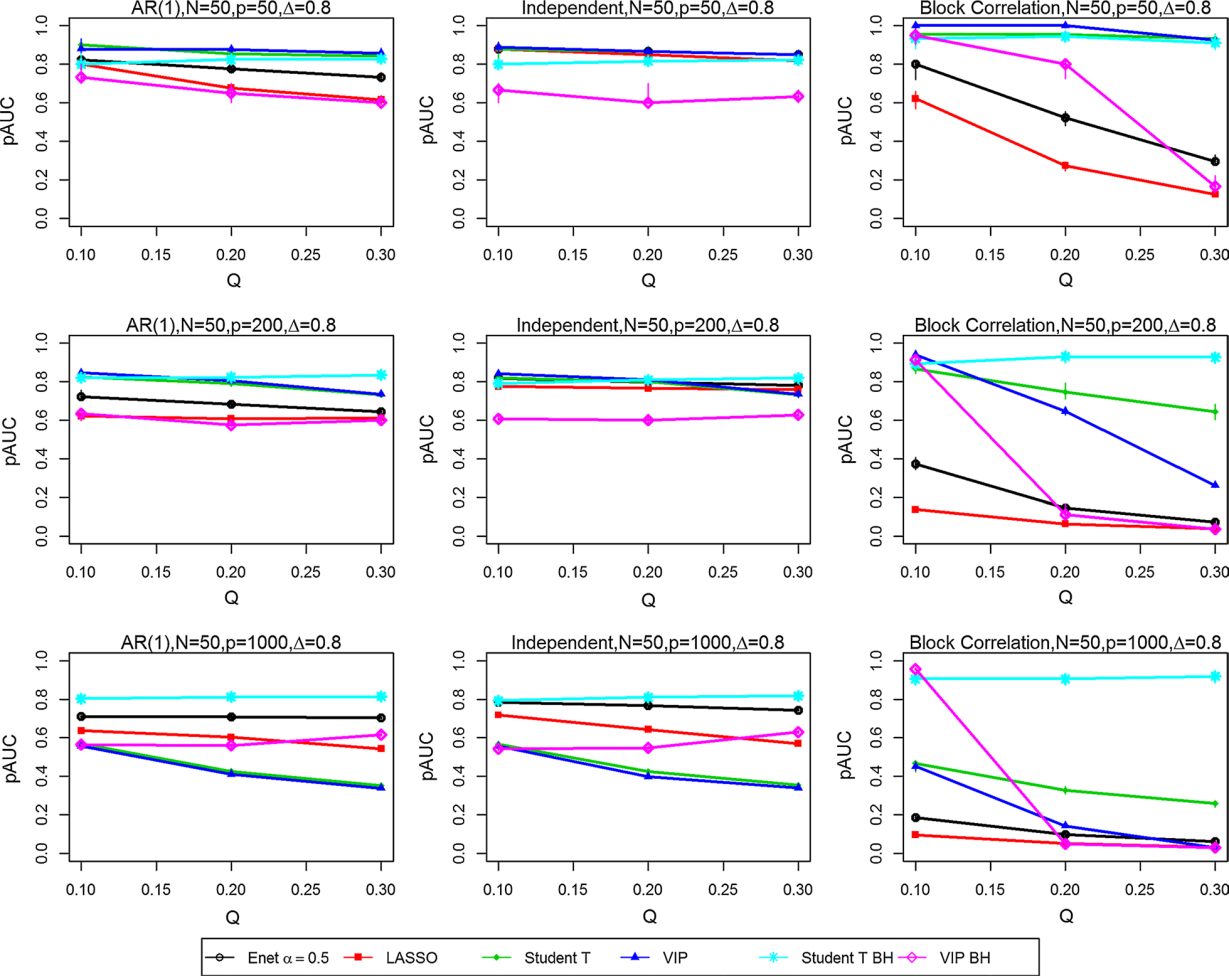
Partial Area Under the ROC curves (pAUCs) as the proportion of significant variables is increased from 0.1 to 0.3. Results are presented for all three correlation structures [independent, AR(1) and block correlation] for the six selection methods [four stability-based methods: Elastic Net (Enet), LASSO, student *t* test, VIP, and two FDR-adjusted methods: student *t* test BH and VIP BH]. The total number N of samples is 50 and the effect size Δ is 0.8. The total number P of variables is 50 (*Top row*), 200 (*Middle row*) and 1000 (*Bottom row*)

**Fig. 4:**
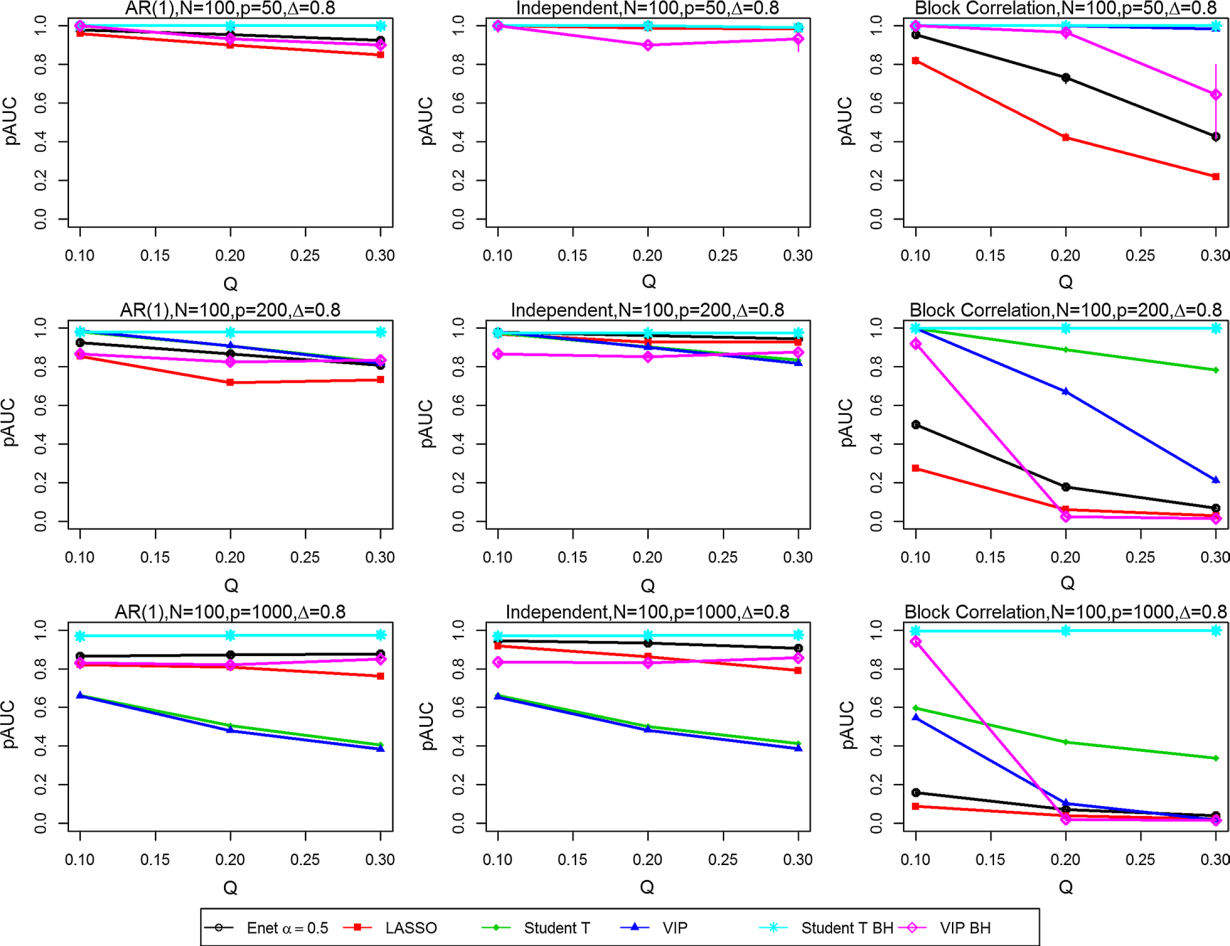
Partial Area Under the ROC curves (pAUCs) as the proportion of significant variables is increased from 0.1 to 0.3. Results are presented for all three correlation structures [independent, AR(1) and block correlation] for the six selection methods [four stability-based methods: Elastic Net (Enet), LASSO, student *t* test, VIP, and two FDR-adjusted methods: student *t* test BH and VIP BH]. The total number N of samples is 100 and the effect size Δ is 0.8. The total number P of variables is 50 (*Top row*), 200 (*Middle row*) and 1000 (*Bottom row*)

Figures 1, 2, 3 and 4 present the simulation study results for the six variable selection methods for various combined sample size N, proportion of true significant variables Q, number of variables P, effect size Δ from treatment group, and correlation structure [i.e., AR(1), independent and block-correlation]. Point estimates represent the median values of pAUC for the 200 simulated datasets for each parameter configuration. Bootstrap sampling was carried out to estimate the 95% confidence intervals (CIs) for the medians of the 200 replicates using algorithm Basic in the R package boot [6]. The widths of the estimated CIs were quite narrow, with an interquartile range of 0.0408, suggesting that the estimated medians did not vary substantially. They are available in tabular form in the Supplementary Materials (https://people.ucalgary.ca/~kakopciu/BootstrapCIs for Simulation Study BMC.xlsx).

In the *independent correlation setting* (centre panels in all figures), all selection methods provided nearly identical results, except when the number of variables and the effect size was high (P = 1000, Δ = 0.8). The study parameters with the greatest effect on the pAUC values were the combined sample size N (Figs. 1 vs. 2, 3 vs. 4) and the effect size Δ (Figs. 1 vs. 3, 2 vs. 4). Doubling the combined sample size from 50 (i.e., 25 per group) to 100 (i.e., 50 per group) increased the pAUC values by at least 0.15 when Δ was 0.4 or 0.8, but had no effect at 0.2 (results not shown). Doubling the effect size (Figs. 1 vs. 3, 2 vs. 4) from 0.4 to 0.8 increased the pAUC values by at least 0.2. pAUC values increased the most when both N and the Δ were increased for all combinations of P and Q compared to when Δ was 0.2 (not all results shown). In the setting with P = 1000 variables and a large effect size (Δ = 0.8), the FDR-adjusted student *t* test and the Elastic Net had pAUCs values that were from 0.15 to 0.4 higher than those for the other four methods.

In the *AR(1) correlation setting* (left panel in all figures), all selection methods provided results that were quite similar to those for the Independent Correlation setting; however, the Elastic Net and LASSO methods had slightly lower pAUC values than in the Independent Correlation setting. For number of variables P = 50 or 200, the FDR-adjusted student *t* test or the student *t* test and VIP obtained using the stability-based approach had pAUC values that were generally higher by at least 0.1 than those for the other methods. As in the Independent Correlation setting, when either the number of variables or effect size was high, the FDR-adjusted student *t* test and the Elastic Net had the highest pAUCs values.

In the *block correlation setting* (right panel in all figures), all six selection methods provided very different results when the effect size was greater than 0.2. The Elastic Net and LASSO consistently ranked the lowest for pAUC values for any P, any Q (i.e., percentage of significant variables), and for any N, and for modest or high Δ. The VIP scores based on an FDR adjustment had higher pAUC values when Q was 0.1, but performed poorly as it increased to 0.2 and 0.3. As Δ was increased, the pAUC values for both unadjusted and FDR-adjusted student *t* tests and the stability-based VIP scores increased substantially: twofold to fourfold when Δ was doubled from 0.2 to 0.4 (results not shown), and from 0.4 to 0.8 when N was 50, but generally only when Q was low (0.1) to modest (0.2). When N was 100, a two-fold increase was observed when Δ was doubled from 0.2 to 0.4 (results not shown), with less dramatic but still substantial increases (0.15 to 0.45) when it was doubled from 0.4 to 0.8. The only scenario where the VIP scores tended to perform worse in the block correlation setting was when Q was high (0.3) and P was at least 200. Both unadjusted and FDR-adjusted student *t* tests were less affected with increasing P and high Q, especially at the greatest effect size (Δ = 0.8). However, when P was high (1000) and Q was greater than 0.1, the FDR-adjusted student *t* test outperformed all other methods.

In the *spiked-in dataset*, the patterns for the pAUC values for the four variable selection methods were also consistent with the model-based simulated datasets when N and Q are small. As shown in Table 2, the pAUC values for the stability-based method were 0.875 for the VIP and both unadjusted and FDR-adjusted student *t* tests, and 0.5 for the LASSO and Elastic Net, indicating that these latter two methods are equivalent to just guessing. In contrast, when the FDR-adjusted *P* values from the VIP and student *t* test were estimated, noticeably lower pAUC values were obtained than for their corresponding methods adopting the stability-based approach. The pAUC from the FDR-adjusted VIP method was 0 versus 0.875 with resampling, and was 0.75 for the FDR-adjusted student *t* test versus 0.875 with resampling. The FDR-adjusted student *t* test selected only six of the eight biomarkers but fewer non-biomarkers, resulting in higher specificity. None of the methods selected Pyroglutamic acid from Solution 2, and most included 1–10 additional metabolites that were not spiked-in. The modest number of replicates can make identification of the spiked-in metabolites more difficult, and may have led to these low pAUC values [24].

**Table 2:** Comparison of results between stability-based and FDR-adjusted methods for the spiked-in data set for four selection methods (true biomarkers in italics font)

Method	Test statistic	Biomarkers selected	pAUC^a^, sensitivity	1-Specificity
Stability (0.5)	VIP	*Gly Ser Thr Ala Val Lys* 20 36 24 13 42 23 41 44 57 19 *Asp*	0.875	0.169
	Student *t* test	*Gly Ser Thr Ala Val Lys* 20 36 13 23 42 41 *Asp* 44 57 24 48	0.875	0.169
	Lasso	*Gly Ala Val *20 23 *Thr* 21	0.5	0.034
	Elastic net	*Gly Ala Val *23 20 *Thr*	0.5	0.051
FDR	Student *t* test *P* values <0.05	*Gly Ser Thr Ala Val Lys* 25	0.75	0.034
	VIP *P* values adj <0.05	–	0	0

^a^ *pAUC* Partial area under the receiver operating characteristic curve (0.2)

## Conclusions

The results from this study add to the literature on variable selection methods in several ways. Multivariate approaches such as VIP scores, which incorporate correlations across the variables, should theoretically outperform simple univariate methods such as the student *t* test when the effect size is low and the significant variables are correlated [25–27]. This was confirmed in this study for the stability-based version of the VIP method in the Block Correlation setting when the effect size was low (Δ = 0.4) but only when the number of measured variables (P = 50) as well as the percentage of significant variables were low (Q < 20%). The FDR-adjusted version of the VIP method also performed well in the Block Correlation setting when the effect size was low (Δ = 0.4) but now when the number of measured variables (P = 200, 1000) was higher and when the percentage of significant variables was very low (Q = 10%). In addition, variable selection based on the student *t* test has been previously shown to perform well in high-dimensional data settings when variables are strongly associated with class label [28]; this was also confirmed in this study when the effect sizes were large (Δ = 0.8) and the number of variables is larger than the sample size (≥100). Chong and Jun [8] also found that selection based on VIP scores performed better than LASSO using similar experimental factors but different performance metrics. The Elastic Net was expected to outperform LASSO when variables are highly correlated [11], which was the case in the simulated data with AR(1) and block correlation, and in the independent correlation setting only when the effect size and number of variables were both large.

**Fig. 5:**
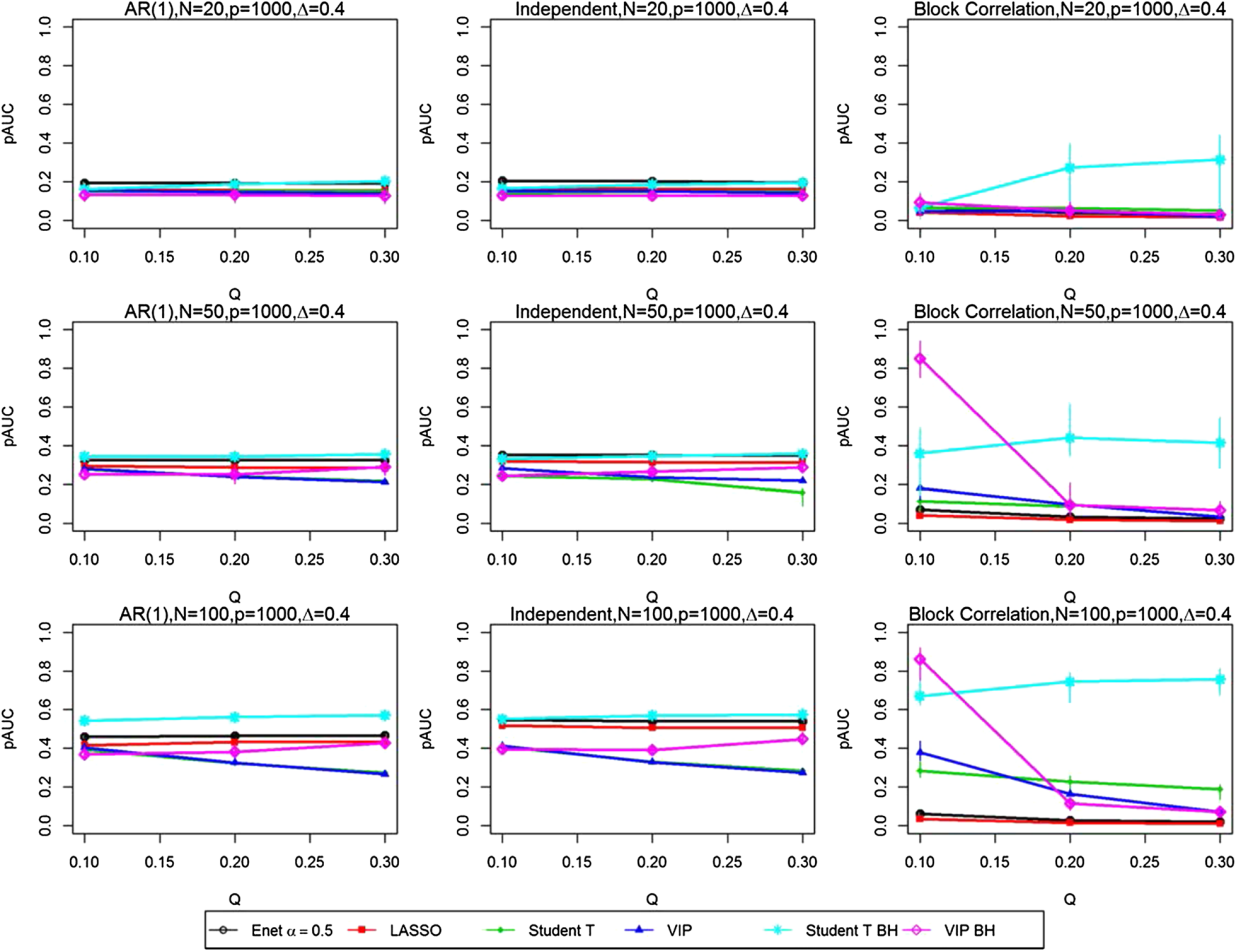
Partial Area Under the ROC curves (pAUCs) as the total number of variables increases from 20 to 100. Results are presented for all three correlation structures [independent, AR(1) and block correlation] for the six selection methods [four stability-based methods: Elastic Net (Enet), LASSO, student *t* test, VIP, and two FDR-adjusted methods: student *t* test BH and VIP BH]. The number P of variables is 1000 and the effect size Δ is 0.4. The total number N of samples is 20 (*Top row*), 50 (*Middle row*) and 100 (*Bottom row*)

Not surprisingly, our finding that increasing the sample size improves prediction was also found to increase classification accuracy in high-dimensional data settings [28, 29]. Implicit in this result for simulated data is that additional heterogeneity is not introduced; that is, all samples are drawn from the same population. To evaluate the effect of a smaller ratio of sample size to number of variables, we examined the setting where the number of variables is high (P = 1000), the effect size is modest (Δ = 0.4) and the sample size per group is modest, low and very low (N/2 = 50, 25 and 10). As we can see in Fig. 5, the effect on the pAUC values of reducing the sample size even further is evident in their general shift downwards, with the single exception of the student *t* test in the Block correlation setting when the number of significant variables is 200 or 300. However, the wide 95% CIs for the student *t* test suggest high variability.

If any correlation is present between the significant variables, using the stability-based VIP scores from a PLS-DA model resulted in better prediction when the effect size was low to modest (0.2–0.4). Chemometrics methods are increasingly being applied in biotechnological processes [30] and the results from this study support using the VIP scores from the projection-based PLS-DA models using the stability-based method when effect sizes are not large and the number of variables is low or modest (≤200).

Finally, the findings from this study provide new results for variable selection methods, especially for the stability-based variable selection approach, which has not previously been extensively evaluated for LASSO and Elastic Net [5, 12]. The stability-based student *t* test and VIP scores outperformed Elastic Net and LASSO in all parameter configurations but not when the effect size and the number of variables were large. In general, the stability-based VIP scores performed similarly to or better than the student *t* test while Elastic Net performed the same as or slightly better than LASSO. When the number of variables was very large, the FDR-adjusted student *t* test performed consistently well.

The objective of this paper was to provide guidance on popular and easily accessible variable selection methods already available in R and readily accessible to any researcher. The R package *BioMark* provides several additional variable selection methods for identifying important variables to classify new observations into one of two groups, including principal components. The stability-based selection approach adopted in this study avoids over-fitting and is robust even when the sample size in each group is small. Our results suggest that using the VIP scores over the other three stability-based variable selection methods, as it generally provides the highest pAUC values. It was the best performing method when the number of variables was low, and especially when the effect size was modest (≤0.4). When there is a large number of variables (*P* = 1000) and block correlation is present, the FDR-adjusted student *t* test performed the best—even in the very-small-sample size setting (10 per group). Thus, it should be the preferred approach in this high variable-to-sample ratio setting. Doubling the sample size from small (50 observations in total) to modest (100 observations in total) tended to yield an increase in the pAUC for any method and any correlation structure with at least some signal in the data (Δ ≥ 0.2). Future research directions should examine ultra high-dimensional data settings to see if similar findings hold and should explore the performance of the regularization methods Elastic Net and LASSO across a wider range of sparse data settings. Finally, extension of the stability-based selection approach to other study designs (e.g., three or more groups) would increase its usefulness and widen its applicability.

## Abbreviations

Ala: alanine
AR(1): autoregressive of order 1
Asp: aspartic acid
AUC: area under the roc curves
GC–MS: gas chromatography–mass spectrometry
Gly: glycine
LASSO: least absolute shrinkage and selection operator
Lys: lysine
NIST: National Institute for Standards and Technology
pAUC: partial area under the receiver-operating curve
PLS-DA: partial least squares regression-discriminant analysis models
Pyr: pyroglutamic acid
RI: retention index
ROC: receiver operating characteristic curves
RSD: relative standard deviation
Ser: serine
Thr: threonine
TMS: trimethylsilyl groups
Val: valine
VIP: variable importance in projection
